# Synthesis and Anti-inflammatory Performance of Newly Cyclizine Derivatives on Adult Male Wistar Rats 

**Published:** 2012

**Authors:** Abbas Ahmadi, Mohsen Khalili, Shahnaz Chavrogh, Babak Nahri-Niknafs

**Affiliations:** a *Department of Chemistry, Faculty of Science, Islamic Azad University, Karaj Branch, Karaj, Iran. *; b *Department of Physiology, Neuroscience and Herbal Medicine Research Center, Shahed University, Tehran, Iran. *; c *Islamic Azad University, Karaj Branch, Karaj, Iran. *

**Keywords:** Cyclizine, Piperazine derivative, H1-antihistamine, Acute and chronic, Anti-inflammation

## Abstract

Cyclizine (1-benzhydryl-4-methyl-piperazine, CAS 82-92-8, CYC, I), a piperazine derivative, belongs to H1 antihistamine group of drugs that shows such pharmacological properties as anti-inflammatory, anti-allergic and anti-platelet effects, similar to other H1-receptor antagonists. In this study, two new tolyl and cumene derivatives of I (1-ethyl- 4-[(*p*-isopropylphenyl) (*p*-tolyl) methyl]-piperazine, II and 1-[3, 4-dichlorophenyl]-4-[[*p*-isopropylphenyl] [*p*-tolyl] methyl]-piperazine, III) were synthesized to investigate their acute and chronic anti-inflammatory activities in formalin and histamine-induced rat paw edema. In addition, the vascular permeability in formalin and histamine-induced paw edema, xylene-induced ear edema, and peritonitis due to acetic acid application into peritoneal cavity were measured. The cotton pellet-induced granuloma model was chosen for inducing chronic inflammation in rats. Findings proved reduction in formalin-induced rat paw edema and vascular permeability (acute inflammation) by I and II at 30 min after the injection. In addition, results in histamine-induced rat paw edema showed anti-inflammatory effects of all drugs started 60 min after the injection as these effects continued for a longer period by II and III comparing to I, as discussed above. In addition, the data on vascular permeability in xylene-induced ear edema and acetic acid-induced to peritoneal cavity confirmed that substitutions on cyclizine molecule were more effective and could decrease the vascular permeability and acute inflammation. However, the results from the cotton pellet-induced granuloma formation in rats revealed that none of the drugs (I-III) were effective to reduce the reactions and intermediates of chronic inflammation.

## Introduction

**Figure 1:**
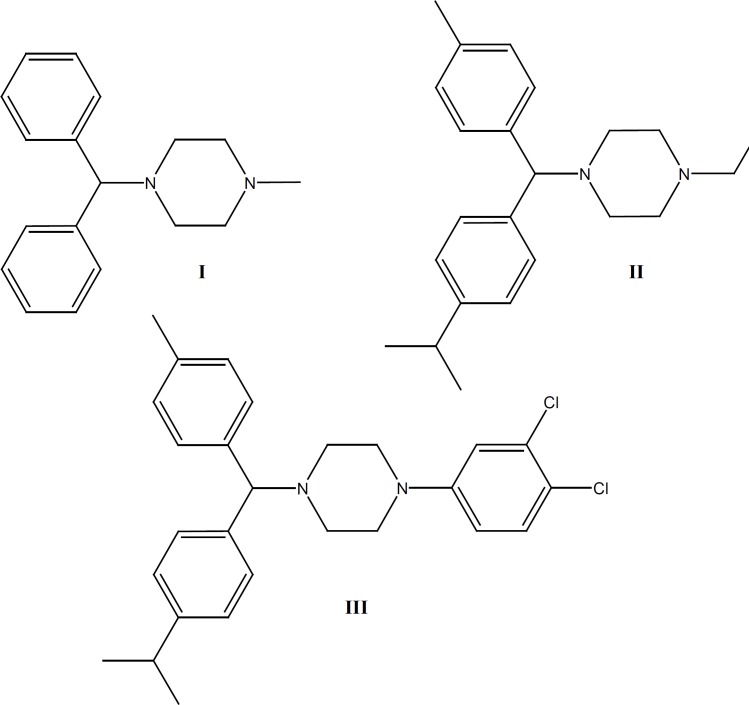
Structure formulas of Cyclizine (Cycl, I), 1-ethyl-4-[(*p*-isopropylphenyl)(*p*-tolyl) methyl]-piperazine (Cycl-1, II) and 1-(3, 4-dichlorophenyl)-4-[(*p*-isopropylphenyl)(*p*-tolyl) methyl]-piperazine (Cycl-2, III

Cyclizine (1-benzhydryl-4-methyl-piperazine, CAS 82-92-8, CYC, Figure 1) is a piperazine derivative which belongs to H1- antihistamine group of drugs (1). In addition to the antihistamine effects, their H1-receptor antagonists show such pharmacological properties as anti-inflammatory and anti-allergic effects, antiplatelet activities, and suppression of respiratory burst of professional phagocytes that are not uniformly distributed among this class of drugs (2-5). 

Most H1-antihistamines exhibit an onset of action within 1 to 2 h after the administration and 24 h duration of action after a single dose. “Maps” for pharmacokinetic/pharmacodynamic relationship in H1-antihistamines define plasma concentration/activity relationship in these medications (6). 

Recently, our knowledge on histamine roles in specific activation or blockade of the receptor subtypes, both in physiology and pathology, has been dramatically increased. Among the four subtypes, histamine H1-receptor has been an attractive target to drug discovery for several years. H1-receptor antagonists have been proved to be effective therapeutic agents in many diseases; hence, they are incorporated into an important class of available drugs (7). 

Inflammation is a disorder involving localized increase in numbers of leukocytes and varieties of complex mediator molecules (8). Prostaglandins are ubiquitous substances that demonstrate and modulate cell and tissue responses involved in inflammation. Their biosynthesis has also been implicated in the pathophysiology of cardiovascular diseases, cancer, colonic adenomas and Alzheimer (9). 

In this paper, two new (II and III) derivatives and intermediates (compounds 1-4) of I were synthesized to evaluate their acute and chronic anti-inflammatory activities using some known pharmacological procedures (10-15). 

## Experimental

*General*

1-methyl piperazine, 1-ethyl piperazine, 1-(3,4-dichlorophenyl) piperazine, Magnesium turning, Diethyl ether, 4-bromo toluene, Benzaldehyde, 4-methyl Benzaldehyde, 4-isopropyl Benzaldehyde, Thionyl chloride and all other chemicals, were purchased from Merck chemical Co. (Darmstadt, Germany). Melting points (uncorrected) were determined with a digital electrothermal melting point apparatus (model 9100, Electrothermal Engineering Ltd., Essex, UK). 1H and 13C NMR spectra were recorded with a Bruker 300 MHz (model AMX, Karlsruhe, Germany) spectrometer (Internal Reference: TMS). IR spectra were recorded with a Thermo Nicolet FT-IR (model Nexus-870, Nicolet Instrument Corp, Madison, Wisconsin, U.S.A.) spectrometer. Mass spectra were also recorded with an Agilent Technologies 5973, Mass Selective Detector (MSD) spectrometer (Wilmigton, USA). Column chromatographic separations were performed over Acros silica gel (No.7631-86-9 particle size 35-70 micrometer, Geel, Belgium). Thirty-two adult male Wistar rats (Pasteur`s Institute, Tehran) weighing 250-300 g were subjected to the pharmacological testing. 

*Preparations (* Figures 1 *-* 3 *)*

*Diphenylmethanol (Benzhydrol) (1)*

**Figure 2:**
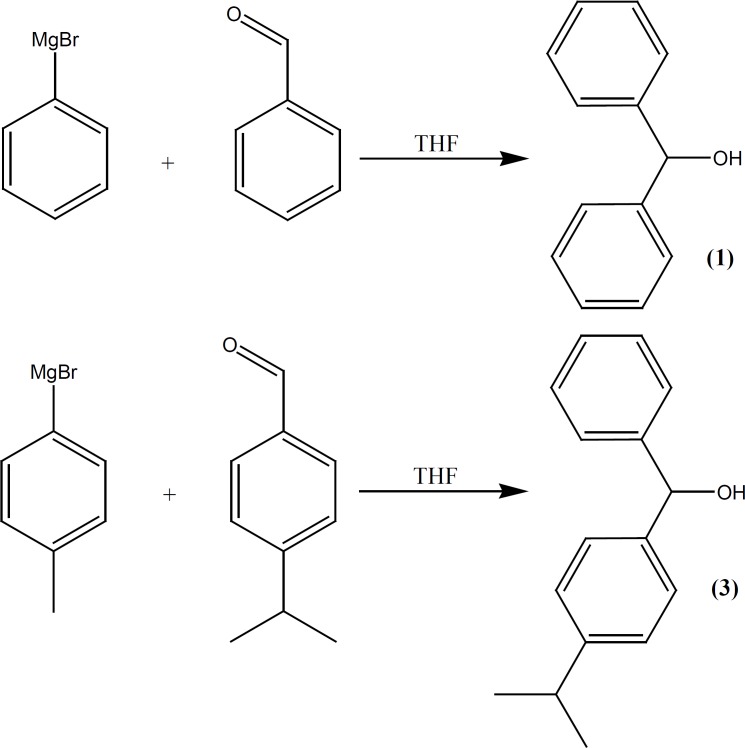
Synthesis of intermediates 1 and 3

This compound was prepared under a published method (16, 17) after some required modification. Phenyl magnesium bromide was added drop-wise to the solution of benzaldehyde (10.6 g, 0.1 mol) in THF (50 mL), (prepared from 15.7 g bromobenzene and 2.43 g of Mg in 50 mL of dry ether) and refluxed for additional 118 h, poured into ice-NH4Cl, before the organic layer was separated, brine-washed, re-extracted with diethyl ether, dried over MgSO4 and evaporated under vacuum. A solid compound (m.p., 64-66°C) was obtained (Figure 2). 

*Chlorodiphenylmethane (Benzhydryl chloride) (2)*

**Figure 3:**
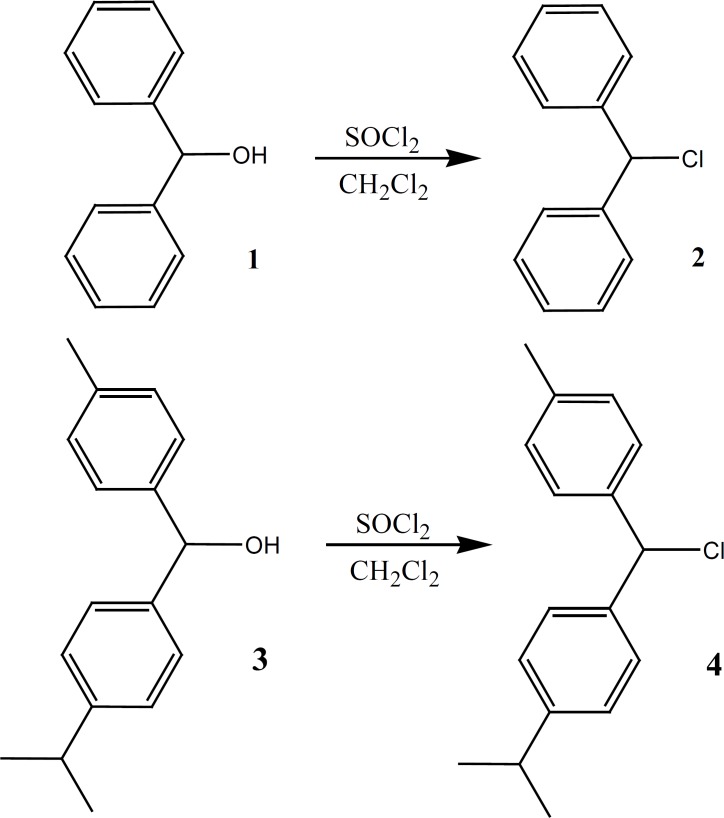
Synthesis of intermediates 2 and 4

This compound was prepared under a published method (16, 17) after some required modification. The alcohol (1, 10.5 g, 0.05 mol) dissolved in dichloromethane (250 mL) and SOCl2 (8 mL, 0.11 mol) was added to the solution. The reaction mixture was refluxed for additional 210 h. The solvent was evaporated under vacuum. An obtained oily brown compound was used in the next step without further purification (highly sensitive to light) (Figure 3). 

*1-Benzhydryl-4-methyl-piperazine (Cyclizine) I*

**Figure 4:**
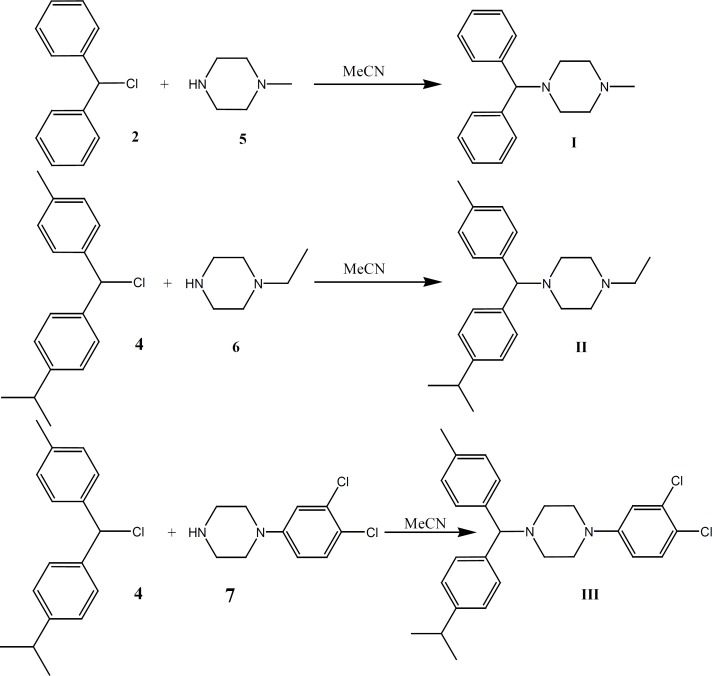
Synthesis of target compounds (I-III).

This compound was prepared under a published method (16-19) after some required modification. The benzhydryl chloride (2, 8 g, 0.04 mol) was dissolved in acetonitrile (100 mL) to which 1-methyl piperazine (5, 15 g, 0.15 mol) was added. The mixture was refluxed for additional 280 h. The solvent was removed under vacuum and the dissolved residue in diethyl ether was water-washed, re-extracted with 10% H2SO4 and neutralized with 10% NaOH. The organic layer was successively water-washed, dried over MgSO4, and evaporated under vacuum to obtain the desired compound (m.p.:105-108) (highly sensitive to light) (Figure 4). The hydrochloride salt for I was prepared with diethyl ether and HCl (highly sensitive to light). 

*(P-isopropylphenyl)(p-tolyl) methanol (3)*

Phenyl magnesium bromide (prepared from 17.09 g bromotoluene and 2.43 g of Mg in 50 mL of dry ether) was added drop-wise to the solution of *p*-isopropyl benzaldehyde (14.82 g, 0.1 mol) in THF (50 mL) and refluxed for additional 125 h. Then poured into ice-NH4Cl and the organic layer was separated, brine-washed, re-extracted with diethyl ether, dried over MgSO4 and evaporated under vacuum. An oily compound was obtained (12.3 g, 56% yield) (Figure 2). 

*IR (KBr): *3401, 2963, 1607, 1512, 1462, 1213, 1170, 1054, 828 cm-1. 

1H N.M.R. (CDCl3) (ppm): 1.31-1.4 (6H, m), 2.02 ( OH, m), 2.34 (3H, s), 3.21-3.31 (1H, m), 5.8 (1H, s), 7.2-7.5 (8H, m). 

13C N.M.R. (CDCl3) (ppm): 23.2, 24.5, 34.1, 77.3, 126.4, 128.05, 129.5, 129.2, 131.4, 148.9. MS: m/z (regulatory intensity): 240 (13). 

*Chloro (p-isopropylphenyl)(p-tolyl) methan (4)*

Alcohol (3, 12.1 g, 0.05 mol) was dissolved in dichloromethane (250 mL) before SOCl2 (8 mL, 0.11 mol) was added to the solution. The reaction mixture was refluxed for additional 230 h and the solvent was evaporated under vacuum. An oily brown compound was obtained (7.1 g, 55% yield) which was used in the next step without further purification (highly sensitive to light) (Figure 3). 

*IR (KBr): *2962, 1611, 1512, 1463, 1421, 1251, 1213, 1182, 1055, 840 cm-1. 

1H N.M.R. (CDCl3) (ppm): 1.31-1.4 (6H, m), 2.34 (3H, s), 3.21-3.31 (1H, m), 6.1 (1H, s), 7.2- 7.5 (8H, m). 13C N.M.R. (CDCl3) (ppm): 23.2, 24.3, 34.5, 77.04, 126.5, 128, 129.4, 129.76, 131.5, 148.3. MS: m/z (regulatory intensity): 258 (16). 

*1-ethyl-4-[(p-isopropylphenyl)(p-tolyl) methyl]-piperazine (Cycl-1) II*

4 (7 g, 0.027 mol) was dissolved in acetonitrile (100 mL) and 1-ethyl piperazine (6, 17.1 g, 0.15 mol). The mixture was refluxed for additional 300 h. The solvent was removed under vacuum and the residue dissolved in diethyl ether, water-washed, re-extracted with 10% H2SO4, neutralized with 10% NaOH. The organic layer was successively water-washed, dried over MgSO4 and evaporated under vacuum to obtain the oily compound (4.9 g, 48% yield) (highly sensitive to light) (Figure 4). 

The hydrochloride salt of I (an oily compound) was prepared using diethyl ether and HCl (highly sensitive to light). 

IR (KBr): 2960, 1607, 1455, 1381, 1289, 1162, 827 cm-1. 

1H N.M.R. (CDCl3) (ppm): 0.96-1.1 (3H, m), 1.31-1.4 (6H, m), 2.34 (3H, s), 2.38-2.49 (10H, m), 3.21-3.31 (1H, m), 5.35 (1H, s), 7.2- 7.5 (8H, m). 

13C N.M.R. (CDCl3) (ppm): 14.4, 23.4, 24.3, 34.5, 51.7, 52.7, 68.5, 77, 126.4, 128.1, 129.2, 129.76, 131.3, 148.9. MS: m/z (regulatory intensity): 336 (9). 

*1-(3, 4-dichlorophenyl)-4-[(p-isopropylphenyl) (p-tolyl) methyl]-piperazine (Cycl-2) III*

4 (7 g, 0.027 mol) was dissolved in acetonitrile (100 mL) before 1-(3,4-dichlorophenyl) piperazine (7, 34.65 g, 0.15 mol) was added. The mixture was refluxed for additional 290 h. The solvent was removed under vacuum and the dissolved residue in diethyl ether was water-washed, re-extracted with 10% H2SO4 and neutralized with 10% NaOH. The organic layer was successively water-washed, dried over MgSO4 and evaporated under vacuum to obtain a yellowish oily compound (6.1 g, 49% yield) (highly sensitive to light) (Figure 4). The hydrochloride salt of I was prepared using diethyl ether and HCl (highly sensitive to light).

IR (KBr): 2961, 2927, 2871, 1610, 1513, 1423, 1421, 1364, 1250, 1055, 840, 755, 698 cm-1.

1H N.M.R. (CDCl3) (ppm): 1.31-1.4 (6H, m), 2.34 (3H, s), 2.7-3 (4H, m), 3.4-3.6 (4H, m), 5.35 (1H, s), 6.9-7.5 (11H, m).

13C N.M.R. (CDCl3) (ppm): 23.4, 24.3, 34.9, 49.8, 51.2, 74.1, 114.2, 116.5, 123.3, 126.4, 128.2, 129.7, 131.7, 134.3, 136.2, 140.4, 147.1, 149.8.

MS: m/z (regulatory intensity): 453 (9).

*Pharmacological methods*

*Animals*

Thirty-two adult male Wistar rats (Pasteur Institute, Tehran), weighing 250-300 g, were randomly housed, three to four per cage, in a temperature-controlled colony room under a 12 h light/dark cycle. Animals were given free access to water and standard laboratory rat chow (Pars Company, Tehran, Iran). All the experiments were implemented between 11 a.m. to 4 p.m. under normal room light and 25°C of temperature. This study was carried out in accordance with the policies provided in the Guide for the Care and Use of Laboratory Animals (NIH) and those in the Research Council of Shahed University of Medical Sciences (Tehran, Iran).

*Anti-inflammatory activities*

*Acute inflammation*

*Formalin-induced rat paw edema*

Thirty-two rats were divided into four groups of eight. The control (normal saline) and treatment groups received I-III compounds (17 mg/Kg, IP), respectively. The administration of drugs was 30 min prior to injection of 50 μL of 3% formalin in the right hind paw subplantar of every rat. The paw volume was initially measured (zero time) and next at 0.5, 1, 2 and 3 h after the formalin injection with a caliper (10). The differences of paw diameter between control and treatment groups were the required data in this study and subjected to statistical analysis.

*Histamine-induced rat paw edema*

The animals were treated in a manner similar to formalin-induced paw edema models. Only histamine (300 μg, 100 μL) was injected into hind paw subplantar surface (12).

*Vascular permeability in formalin and histamine-induced paw edema*

The procedure was similar to Miles and Miles (1952) method (13). Treatment and induction of edema in the present experiment was the same as formalin or histamine models mentioned above. Thirty min after the formalin or histamine injection, the animals received intravenous injection of Evans Blue dye (30 mg/Kg). Thirty min after Evans Blue dye injection, the rats were anesthetized with CO2 inhalation and eventually sacrificed. The inflamed formalin or histamine paws were cut from the wrist region and sectioned to some pieces. The paw pieces were stored in mixture of acetone and sodium sulfate (1%) at a ratio of 3/1 at room temperature under 24 h shaking (IKA-Vibrax, Germany). The mixed solution plus paw sections were centrifuged before their supernatants were collected and their absorbance at 590 nm were measured (Spectronic 20, Germany) as scores of inflammation.

*Vascular permeability in acetic acid-induced to peritoneal cavity*

Tests drugs or vehicle were administered to animals. Thirty minutes later, every rat was given an intravenous injection of Evans Blue solution (30 mg/Kg) followed by an intraperitoneal injection of 0.7% acetic acid at 10 mL/Kg (The method was introduced by Whittle (1964)) (14). Rats were sacrificed by cervical dislocation 30 min after the acetic acid injection. The animals› peritoneal cavity was washed three times with a total of 10 mL saline. Saline washes from the same animal were combined and centrifuged for 10 min at 581×*g *in a table top centrifuge (Sigma-4-10, Germany). Supernatants were collected and their absorbance at 590 nm was measured with a spectrophotometer (Spectronic 20 Genesys, USA). The amount of Evans Blue extruded into the peritoneal cavity was estimated from a standard curve.

*Vascular permeability in xylene-induced ear edema*

The present experiment followed the previously described method (14). Only for inducing ear edema, xylene (0.03 mL) was topically applied to both surfaces of right ears. Ear disks of 8.0 mm in diameter were punched out, sectioned to some pieces and finally subjected to Evans Blue extraction measurement methods for acetic-acid peritonitis.

*Chronic Inflammation*

*Cotton pellet-induced granuloma formation*

The cotton pellets-induced granuloma in rats was examined using the method of D›Arcy *et al*. (1960) (15). The control and treatment groups were anaesthetized (Ketamine 100 mg/Kg), before the sterile cotton pellets weighing 30 ± 1 mg were implanted subcutaneously into both sides of the groin region in every rat. All the animals received the vehicle (saline) or drugs for seven consecutive days after the day of cotton pellet implantation. On the 8th day, the animals were anaesthetized. The pellets and the granuloma tissues were carefully removed and cleaned from extraneous tissues. The wet pellets were weighed and dried in an oven at 60°C for 24 h to constant weight. Next, the dried pellets were weighed again. Increment in the dry weight of the pellets was taken as a measure of granuloma formation.

## Results

*Chemistry*

Cyclizine **(**1-benzhydryl-4-methyl-piperazine, Cycl, I), and new analogues of it (Cycl-1 II, 1-ethyl-4-[(*p*-isopropylphenyl)(*p*-tolyl) methyl]-piperazine) and Cycl-2, III, 1- (3, 4-dichlorophenyl)-4-[(*p*-isopropylphenyl)(*p*-tolyl) methyl]-piperazine) were synthesized by the reaction of substituted benzhydryl chloride (2 and 4) reagents and piperazine compounds (5-7). As methyl and isopropyl groups on the aromatic rings had high electron donating characters, they induced more electron density in the molecules. Moreover, changing methyl by ethyl and 3, 4-dichlorophenyl piperazine generated the pronounced effects on the new drug’s activity. While some known procedures were applied to synthesize the compounds I, 1 and 2, the required modifications were applied (16-19).

Spectroscopic data (IR, 1H and 13C NMR, Mass) confirmed the structure of the compounds 3, 4, II and III. The melting points for the known compounds also confirmed their identity. The purity of every compound was checked by TLC using ethyl acetate-hexane as the eluent.

*Pharmacology*

*General consideration*

Mortality (number of death), morbidity (defined as any abnormal condition or behavior due to a disorder), irritability (a condition of aggressiveness or increased response to handling) and other related abnormal states were observed in experimental animals. However, the motor coordination index (measured by Rota-rod apparatus, Harvard, UK) did not indicate any significant differences among the treated rats.

*Acute inflammation*

*Formalin-induced rat paw edema*

**Figure 5:**
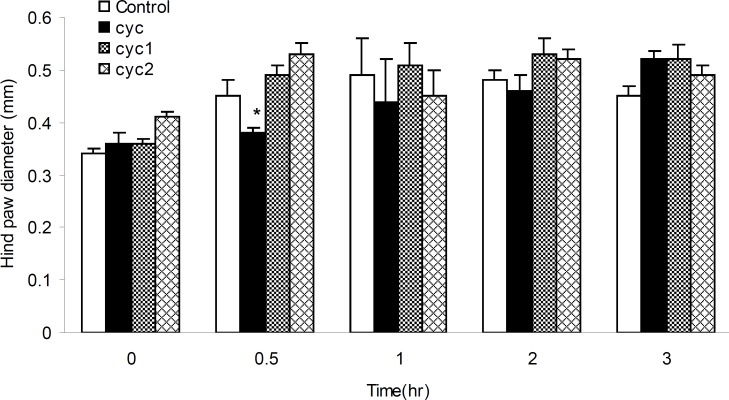
Anti-inflammatory effects of I-III in formalin-induced rat paw edema. Edema was measured in 0, 0.5, 1, 2 and 3 h after the formalin injection. Bars show mean ± SEM of paw diameter. *: p < 0.05 compared with control. (n = 12).

The anti-inflammatory activities of I-III measured at the dose of 17 mg/Kg b.w against the acute paw edema induced by formalin, was summarized in Figure 5. It was found that, I could produce significant (p < 0.05) anti-inflammatory activity (15.55%), 30 min after the formalin injection. Furthermore, III could produce anti-inflammatory activities within 1 h after the formalin injection compared to control and II groups.

*Histamine-induced rat paw edema*

**Figure 6:**
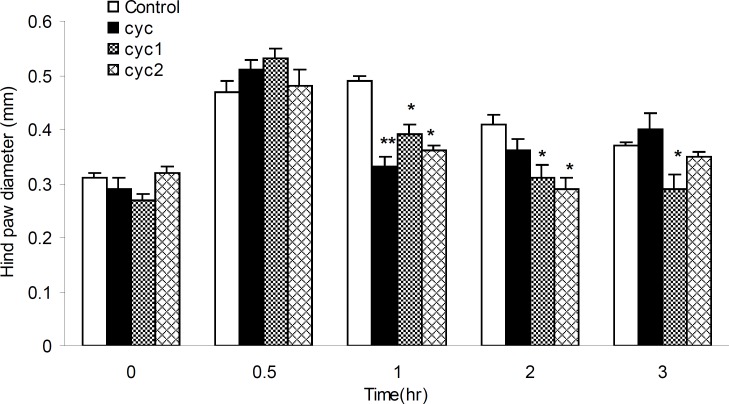
Anti-inflammatory effects of I-III in histamine-induced rat paw edema. Edema was measured in 0, 0.5, 1, 2 and 3 h after the histamine injection. Bars show mean ± SEM of paw diameter. *: p < 0.05, compared with control. (n = 12). **: p < 0.01 compared with control (n = 12).

The anti-inflammatory effects of I-III against acute paw edema induced by phlogistic agent histamine were shown in Figure 6. All drugs showed significant anti-inflammatory effects, 32.65, 20.30 and 26.53%, respectively, within 1 h after the histamine injection (p < 0.01 and 0.05) compared to the control groups. Besides, newly synthesized drugs (II and III) dramatically decreased the inflammation 2 and 3 h after the histamine application (29.26%) and more prominent antiphlogistic effect was seen for I in 2 and 3 h after the histamine injection, 24.39 and 21.62%, respectively (p < 0.05). 

*Vascular permeability in formalin and histamine-induced paw edema*

Data in Table 1 revealed that, I and II could eliminate dye extrusion (edema) from plantar surface of paws 40.00 and 65.71% respectively in the histamine-induced inflammation model (p < 0.01). Moreover, compound II showed more anti-inflammatory effects compared to compound I (p < 0.05) (Table 1). In formalin-induced edema method, extensive antiphlogistic effects from compound II were determined close to 66.66% (p < 0.001). 

**Table 1:** Effect of I-III on formalin and histamine paw inflammations, xylene-induced ear edema and acetic acid-induced peritonitis

**Evans blue dye extracted solution light absorbance (inflammation score) **					
**Inflammation induced methods **		**Histamine injection to hind paw **	**Formalin injection to hind paw **	**Xylene injection to ear **	**Acetic acid injection to peritoneal **
**Animal groups test **	**Control **	0.35 ± 0.08	0.30 ± 0.06	0.21 ± 0.02	0.49 ± 0.07
	**I **	0.21 ± 0.04 **	0.19 ± 0.03	0.19 ± 0.04	0.37 ± 0.07
	**II **	0.12 ± 0.01 **$	0.10 ± 0.04 **	0.11 ± 0.03 **$$	0.22 ± 0.03 *
	**III **	0.25 ± 0.06	0.12 ± 0.04	0.09 ± 0.01 **$$	0.27 ± 0.07 *

The numbers show mean ± SEM of paw, ear and peritoneal solutes extract light absorption. *, **p < 0.05, 0.01 and $, $$ p < 0.05, 0.01 show the difference with control and I animal group test in each series of experimental method. n = 12 in each group of test.

*Vascular permeability in xylene-induced ear edema*

As shown in Table 1, II and III exhibited significant inhibitory effects on xylene-induced ear edema compared to control and animal groups I (p < 0.01). However, the inhibition rate of edema in comparison with control and I was calculated 46.61 and 42.10% for II and 57.14 and 52.26% for III, respectively. 

*Vascular permeability in acetic acid-induced to peritoneal cavity*

As shown in Table 1, II and III could inhibit acetic acid-induced dye extrusion into the peritoneal cavity by 55.1 and 44.89%, respectively. Statistics proved these effects as significant compared to the control rats (p < 0.05). 

*Chronic inflammation*

*Cotton pellet-induced granuloma formation in rats*

**Figure 7:**
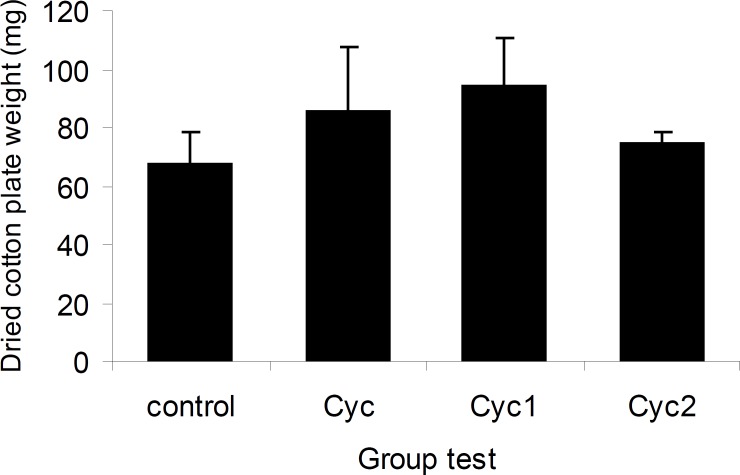
Chronic anti-inflammatory effects of I-III in cotton plate implantation model. There was no significant effect between the control and treatment animals. Bars show mean ± SEM cotton dry weight (n = 12).

The effects of I-III on cotton pellet-induced granuloma formation were shown in Figure 7. The weight gain of cotton pellet from 67.10 ± 11.58 g in control rats to 85.10 ± 22.34 g for I, 94.14 ± 16.07g for II and 74.20 ± 4.26 g for III, respectively, were not statistically significant. 

*Statistical analysis*

Data from parameter levels assessment were expressed as means ± SEM. One-way analysis of variances (ANOVA) was calculated followed by the post-hoc Turkey test in p-value < 0.05. 

## Discussion

Histamine is an important chemical mediator that may cause inflammation, vasodilation, increased vascular permeability, decreased peripheral resistance, airway smooth muscle contraction, and itching sensory nerve stimulation (20). 

Evidences suggest that H1-antihistamines generate anti-inflammatory and immune- modulatory activities that are too extensive to be explained by H1-receptor blockade (21, 22). There is also some evidence that H1-antihistamines may affect the upregulation of the number and function of *β*2-receptors. The exact mechanism of the anti-inflammatory effects of H1-antihistamines is barely clear. Anti-inflammatory effects may occur as a consequence of their ability to influence the activation of genes that regulate the production of pro-inflammatory cytokines and adherent proteins (23). In addition, the cationic amphophilic nature of most H1-antihistamines suggests that they may form an association with cell membranes and thereby, inhibit the calcium binding and the membrane-associated enzymes activities (24).

Synthesis of cyclizine (I) was reported by Baltzly *et al*. (16), while its antihistaminic activities were discovered by Castillo *et al. *(25). In this study, two new derivatives of I that induced some changes in substitutions in phenyl and piperazine moieties were synthesized. The acute and chronic anti-inflammatory effects of these compounds were evaluated by standard pharmacological methods (10-15).

In formalin-induced rat paw edema (diameter change measuring, Figure 5 or vascular permeability, Table 1), the results showed decreased paw edema and vascular permeability (acute inflammation) for I and II, 30 min after the injection. Moreover, the results of histamine-induced rat paw edema (diameter change measuring, Figure 6 or vascular permeability, Table 1), proved the anti-inflammation effects of all drugs started 60 min after the injection. These effects were continued, however, for longer periods of time by II and III compared to I, as argued above (for II). Therefore, it could be concluded that substituting phenyl by tolyl and cumene rings, methyl by ethyl and 3, 4-dichlorophenyl piperazine in II and III decreased paw edema and vascular permeability.

In addition, the obtained data from vascular permeability in xylene-induced ear edema and acetic acid-induced to peritoneal cavity confirmed that these substitutions on cyclizine molecule were more effective and could decrease the vascular permeability and acute inflammation. But the results from the cotton pellet-induced granuloma formation in rats showed that none of the drugs (I-III) were effective to reduce the reactions and intermediates of chronic inflammation.

## Conclusion

Addition of methyl and isopropyl groups in phenyl rings with high electron donating, and substituting the methyl group with ethyl and 3,4-dichlorophenyl piperazine group in newly synthesized drugs, generated noticeable effects in decreasing the acute inflammation and the prominent antiphlogistic results. These effects by the new drugs might be related to the reduction of vascular permeability mechanism(s) or more antagonistic effects on H1 histamine receptors.
